# Insights into *ZmWAKL* in maize kernel development: genome-wide investigation and GA-mediated transcription

**DOI:** 10.1186/s12864-023-09849-6

**Published:** 2023-12-11

**Authors:** Kun Hu, Qiao Dai, Babatope Samuel Ajayo, Hao Wang, Yufeng Hu, Yangping Li, Huanhuan Huang, Hanmei Liu, Yinghong Liu, Yayun Wang, Lei Gao, Ying Xie

**Affiliations:** 1 National Demonstration Center for Experimental Crop Science Education, College of Agronomy, Sichuan Agricultural University, Chengdu, 611130 China; 2 College of Life Science, Sichuan Agricultural University, Ya’an, 625014 China; 3 Maize Research Institute, Sichuan Agricultural University, Chengdu, 611130 China; 4 State Key Laboratory of Crop Gene Exploration and Utilization in Southwest China, Sichuan Agricultural University, Chengdu, 611130 China; 5 Sinograin Chengdu Storage Research Institute Co.Ltd, Chengdu, 610091 China

**Keywords:** Genome-wide identification, Wall associated kinase, Gibberellic acid, Transcriptional factor, Maize Kernel

## Abstract

**Background:**

The functional roles of the Wall Associated Kinase (WAK) and Wall Associated Kinase Like (WAKL) families in cellular expansion and developmental processes have been well-established. However, the molecular regulation of these kinases in maize development is limited due to the absence of comprehensive genome-wide studies.

**Results:**

Through an in-depth analysis, we identified 58 maize *WAKL* genes, and classified them into three distinct phylogenetic clusters. Moreover, structural prediction analysis showed functional conservation among *WAKL*s across maize. Promoter analysis uncovered the existence of *cis*-acting elements associated with the transcriptional regulation of *ZmWAKL* genes by Gibberellic acid (GA). To further elucidate the role of *WAKL* genes in maize kernels, we focused on three highly expressed genes, viz *ZmWAKL38*, *ZmWAKL42* and *ZmWAKL52*. Co-expression analyses revealed that their expression patterns exhibited a remarkable correlation with GA-responsive transcription factors (TF) TF5, TF6, and TF8, which displayed preferential expression in kernels. RT-qPCR analysis validated the upregulation of *ZmWAKL38*, *ZmWAKL42*, *ZmWAKL52*, TF5, TF6, and TF8 following GA treatment. Additionally, *ZmWAKL52* showed significant increase of transcription in the present of TF8, with *ZmWAKL52* localizing in both the plasma membrane and cell wall. TF5 positively regulated *ZmWAKL38*, while TF6 positively regulated *ZmWAKL42*.

**Conclusions:**

Collectively, these findings provide novel insights into the characterization and regulatory mechanisms of specific *ZmWAKL* genes involved in maize kernel development, offering prospects for their utilization in maize breeding programs.

**Supplementary Information:**

The online version contains supplementary material available at 10.1186/s12864-023-09849-6.

## Background

The plant cell wall plays crucial roles in furnishing structural support and protection, setting it apart from its animal cell counterparts. It also plays a crucial role in determining cell size and shape, thus influencing plant development. Thus, questions arise regarding the regulation of cell wall synthesis and expansion [1]. In addition to protecting plant cells against external stresses and providing structural integrity, the cell wall contributes to plant development by influencing processes like cell elongation, division, tissue differentiation, and organ formation. Communication between the cell wall and other cellular components, such as the plasma membrane and cytoplasm, is crucial for coordinating these processes and determining cell size and shape [2].

The WAK gene has emerged as a key regulator of cell elongation and cell wall synthesis, thereby affecting plant morphogenesis. *WAK* genes influence various plant development processes, particularly those related to plant growth and shape [3–5]. For instance, studies on rice have demonstrated that a specific *WAK* gene from this crop is involved in plant development. Suppression of its expression leads to dwarf plants with reduced leaf size, flag-leaves, internodes, and panicles [6]. Similarly, suppression of *WAK4* gene expression in *Arabidopsis* results in altered morphology, including reduced cell elongation, leading to small rosette leaves, condensed inflorescence stems, short siliques, unopened miniature flowers, and short primary roots [1, 7].

Further investigation reveals that *WAK* belongs to the receptor-like kinase (RLK) superfamily. RLKs share a common structural feature: a carboxyl-terminal cytoplasmic region with an active Ser/Thr kinase domain. WAKs also possess an amino-terminal extracellular region containing various motifs, including a highly conserved epidermal growth factor (EGF) repeat, Ca^2+^-binding EGF domains, and EGF2-like domains [8]. The presence of the EGF domain in WAK is closely associated with cell wall synthesis and plays a significant role in cell elongation [3]. There is another group of genes called WAKL family, which exhibits a similar structure to WAK, incorporating both the RLK region and at least one EGF domain. The WAK/WAKL family includes numerous well-studied genes in various plant species, such as cotton, barley, apple, and rice [9–11].

The *WAK* gene family has emerged as a critical player in various aspects of plant biology, including cell wall synthesis, cell elongation, morphogenesis, and plant growth and development [3–5]. Extensive research has been conducted to unravel their functional significance in different plant species. For instance, in maize, a specific member of the *WAK* family, *ZmWAK-RLK1* (*Htn1*), has been identified as a regulator of disease resistance against the fungal pathogen *Exserohilum turcicum*, causing head smut disease [12–14]. The discovery of this gene's involvement in disease resistance raises possibilities for enhancing resistance in maize through genetic improvement strategies. While the role of the *WAK* gene family in disease resistance has been extensively studied, further exploration of their broader functions and underlying molecular mechanisms is imperative. Genome-wide analyses, functional characterization, and exploration of interactions with other genes and signaling pathways are essential for a deeper understanding of the diverse roles played by the *WAK* gene family in plant biology.

Plant hormones, including abscisic acid (ABA), methyl jasmonate (MeJA), salicylic acid (SA), and gibberellic acid (GA), regulate various developmental processes in plants. They have diverse functions in shaping plant structures, including roots, flowers, stems, and leaves. Furthermore, these hormones play significant roles in fruit development, seed germination, growth regulation, plant longevity, and programmed cell death [15–17]. GA, in particular, is important in regulating cell elongation, division, and overall plant growth. GA can change cell shape by improving the extensibility of cell wall [18–21]. The effects of GA on cell elongation and division vary depending on the tissue and developmental stage. In cotton, increased levels of GA lead to enhanced cell wall synthesis, resulting in thicker cell walls, improved fiber quality, and increased cellulose content [22]. In wheat and maize, GA affects kernel development [23–25]. By introducing GA-sensitive dwarf genes in wheat, plant height is reduced, leading to increased kernel number and size, ultimately enhancing grain yield [23]. Similarly, GA application during the 4–5 leaf stage in maize significantly improves yield attributes [26]. The overlap in function between GA and *WAK/WAKL* in cell elongation suggests a potential interaction or regulatory relationship between them.

Plant hormones, including GA, have been shown to modulate gene expression in various physiological processes. The interaction between GA and the *WAK/WAKL* gene family has been investigated in different crops. In potatoes, GA-responsive elements were identified in several *WAK/WAKL* genes. GA treatment significantly upregulated the expression of the *StWAK12* gene in potato plants [27]. In cotton, GA-responsive elements were found in *GhWAK* genes, and GA treatment induced the expression of most *GhWAK* genes [28]. However, it remains unclear whether GA affects *WAKL* gene expression in maize. Further research is required to determine the specific effects of GA on *WAKL* gene expression in maize, as well as the underlying mechanisms involved.

In conclusion, our study provides a comprehensive investigation of the *WAKL* gene family in maize, with a focus on their regulation by GA and associated transcription factors (TFs). We identified 58 *ZmWAKL* genes classified into three groups. Remarkably, eight *ZmWAKL* genes exhibited high expression during early kernel development. Notably, GA treatment significantly upregulated the expression of *ZmWAKL38*, *ZmWAKL42* and *ZmWAKL52*. These findings offer valuable insights into the functional characterization of the maize *WAKL* gene family and shed light on the involvement of GA-responsive transcription factors in regulating *ZmWAKL38*, *ZmWAKL42*, and *ZmWAKL52*. Future studies in this field will expand our understanding of the intricate regulatory mechanisms underlying GA-mediated *ZmWAKL* gene expression in maize.

## Results

### Genome-Wide Identification and Chromosome Localization Analysis of *WAKL* Genes in Maize

Through an extensive genome-wide analysis using the HMM method, we successfully identified 165 potential loci responsible for encoding WAKL proteins in maize. These findings align with previous studies conducted on *Arabidopsis thaliana*, *Oryza sativa*.L, and *Hordeum vulgare*.L, confirming the presence of two conserved domains within the maize WAKL proteins: EGFs (EGF repeat, Ca^2+^-binding EGF domain, and EGF2-like domain) (File S1: Table S1.3) and a protein kinase domain (File S1: Table S1.1) [10, 29, 30].To validate the presence of these conserved domains, we subjected the protein sequences of the 165 candidates to diagnostic domain analysis utilizing CDD, Pfam, and ClustalW. As a result, we successfully identified 58 sequences as *ZmWAKL* genes and assigned them names based on their respective chromosome locations.

Detailed information regarding the characteristics of ZmWAKL proteins can be found in Table S2. The analysis revealed significant variability in protein length, ranging from 342 amino acids for ZmWAKL25 to 1328 amino acids for ZmWAKL02. Correspondingly, the molecular weights ranged from 38.03 kDa for ZmWAKL25 to 147.35 kDa for ZmWAKL02. The predicted isoelectric points (pI) of ZmWAKLs varied between 4.96 (ZmWAKL17) and 8.96 (ZmWAKL43), indicating an overall weakly acidic nature with an average pI of 6.40. Moreover, the calculated GRAVY values were negative, suggesting the hydrophilic nature of the ZmWAKL proteins. Although the majority of *ZmWAKL* genes exhibited a single transcript variant, approximately 20 genes demonstrated the presence of multiple transcript variants, indicating the presence of transcriptomic complexity within this gene family. Particularly noteworthy were *ZmWAKL44*, which exhibited 17 transcripts, and *ZmWAKL46*, which showed 13 transcripts. These findings exemplify the intricate transcriptional regulation and potential functional diversity of these genes.

### Phylogenetic and molecular evolution analysis of *WAKL* genes in maize

**Fig. 1:**
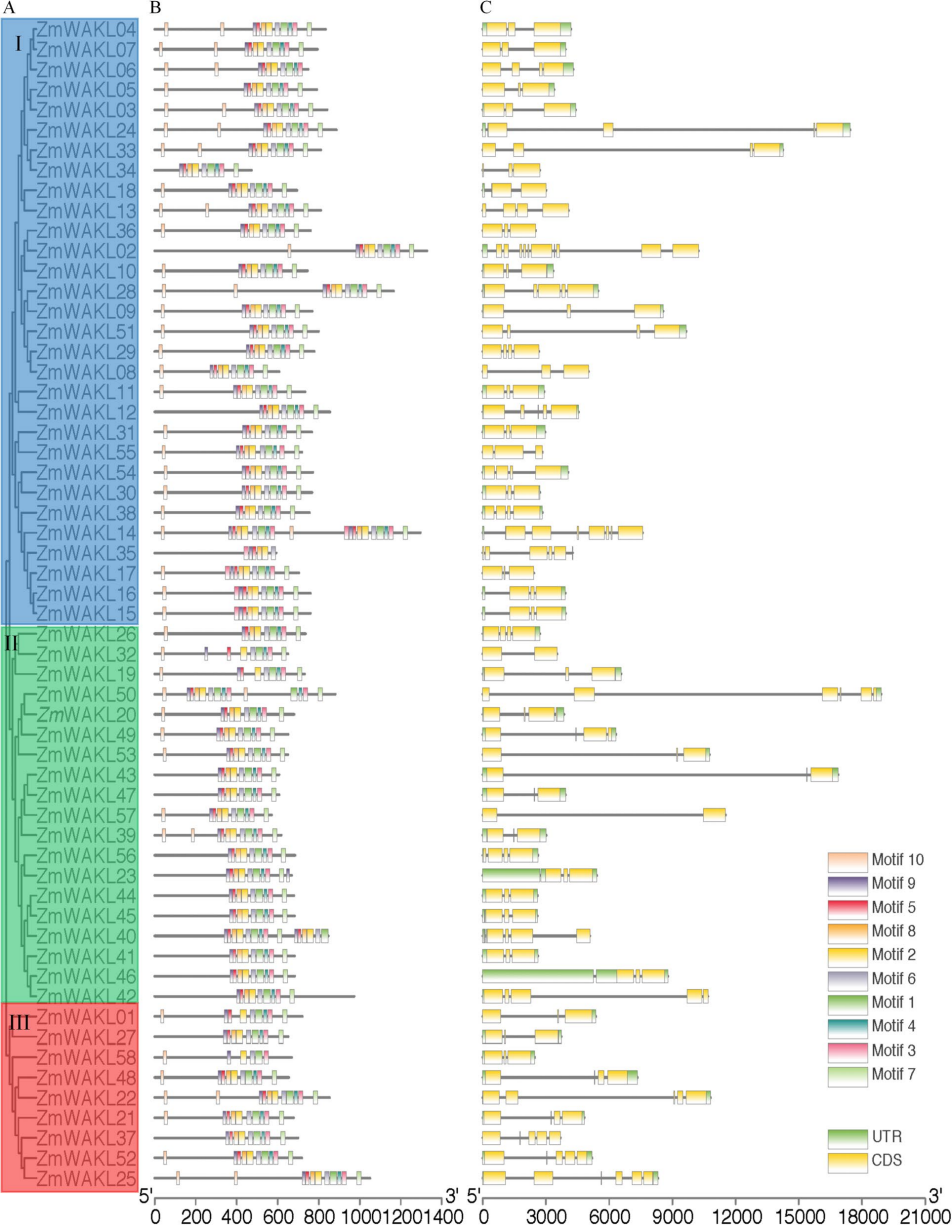
Phylogenetic analysis and structural features of *ZmWAKL* genes. **A** Phylogenetic tree of 58 ZmWAKL proteins were classified into 3 groups and were marked with different color. **B** Conserved motifs of ZmWAKLs, identified using the MEME database and complete amino acid sequences. **C** Exon/intron organization of *ZmWAKL* genes. Exons are depicted as yellow boxes, black lines indicate introns, and UTR regions are represented by green boxes. The information of the 10 motifs were listed in File S1: Table S1.2. The scale at the bottom represents the inferred length of exons

The phylogenetic analysis of 58 ZmWAKL proteins in maize identified three main clusters: Group I, Group II, and Group III, with 29, 19, and 10 proteins, respectively (Fig. S1 and Fig. 1A). Gene structure analysis confirmed that genes within the same group and neighboring clades displayed similar structural patterns. For instance, Group I members, like *ZmWAKL15* and *ZmWAKL16*, shared three exons, reflecting their close phylogenetic relationship. However, slight variations in exon–intron distribution were observed among different phylogenetic groups, resulting in a range of 2 to 5 exons within the *ZmWAKL* gene family (Fig. 1C).

To explore the phylogenetic relationships and structural diversity of *ZmWAKL* genes, we used the MEME online server to identify conserved motifs (File S1: Table S1.1). Ten motifs associated with the wall-associated kinase or protein kinase domain were found among ZmWAKL proteins (Fig. 1B). Remarkably, almost all Group I members exhibited all 10 motifs (File S1: Table S1.2), including the widely-present WAK motif. This motif was predominantly located in the N-terminal region of most *ZmWAKL* genes, indicating a conserved distribution pattern within the family. Furthermore, closely related proteins in adjacent clades of the phylogenetic tree displayed similar motif structures. For example, *ZmWAKL28* and *ZmWAKL09*, both from Group I, shared all 10 motifs, including the STKc_IRAK motif, which is exclusive to genes in adjacent clades and the same group. EGF motifs were present in some ZmWAKL proteins, consistent with their definition (File S1: Table S1.3). This finding aligns with previous research by Li [31]. The combined evidence from phylogenetic analysis, conserved motif distribution, and gene structure similarity strongly supports the conservation and reliability of group classification in *ZmWAK*L genes.

### Chromosome localization and synteny analysis of *ZmWAKL* genes

**Fig. 2:**
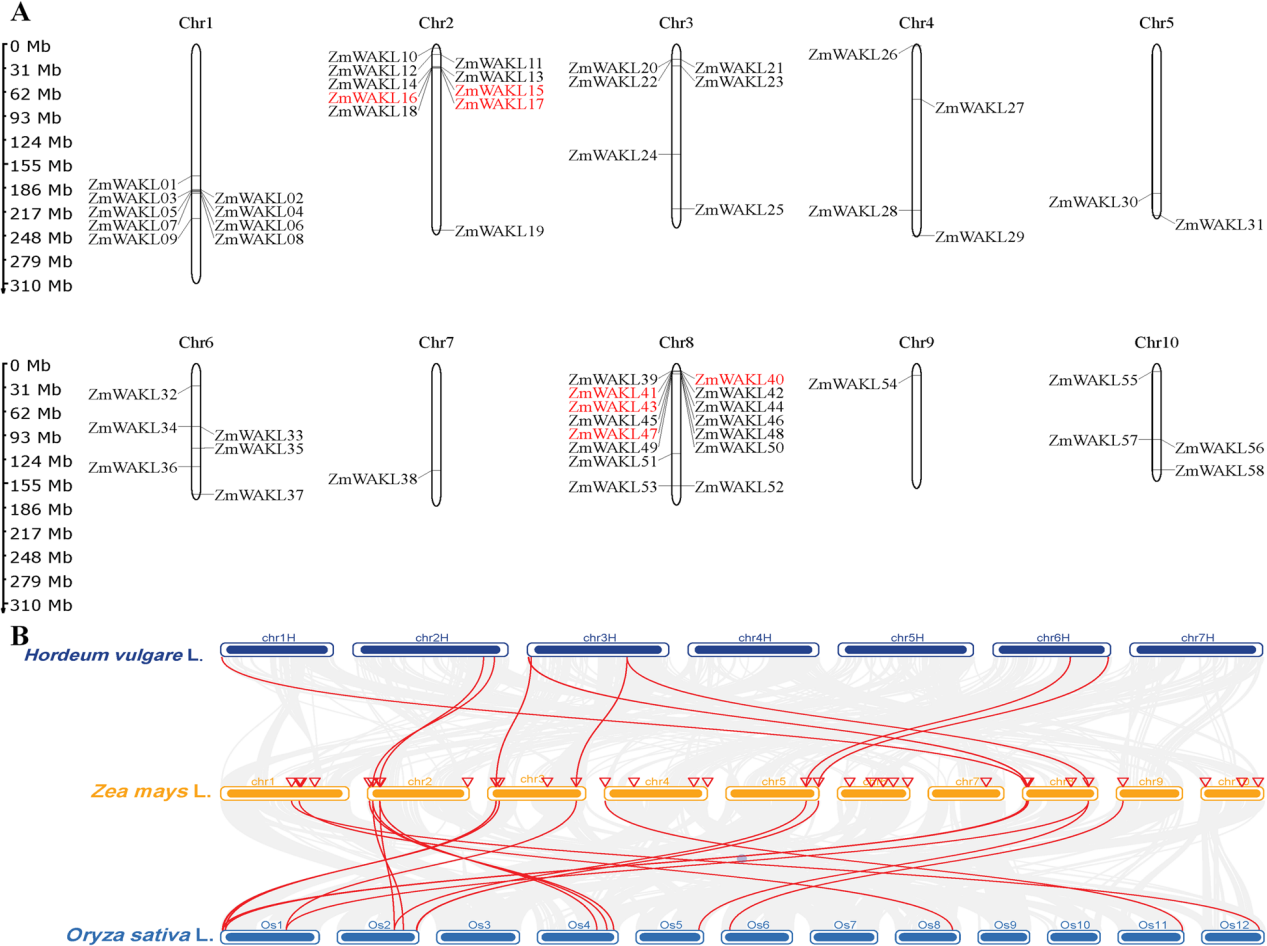
Chromosomal mapping and comparative synteny analysis of *ZmWAKL* genes. **A** Chromosome localization and gene duplication patterns of *ZmWAKL* genes are illustrated. The figure displays the positions of *ZmWAKL* genes on maize chromosomes, with tandem duplicated genes highlighted in red font. The left-side scale bar represents the length of maize chromosomes in megabases (Mb). **B** Comparative synteny analysis of *WAKL* genes across *Zea mays* L. (maize), *Hordeum vulgare* L. (barley), and *Oryza sativa* L. (rice)

The chromosomal positions of the 58 *ZmWAKL* genes were determined based on their starting positions, with their names assigned accordingly. Analysis of the distribution across maize chromosomes revealed a non-uniform pattern (Fig. 2A). Chromosome 8 contained the highest number of *ZmWAKL* genes (15 genes), followed by chromosomes 2 and 1 with 10 and 9 genes, respectively. Chromosomes 3, 6, 4, and 10 harbored 6, 6, 4, and 4 *ZmWAKL* genes, respectively, while chromosomes 7 and 9 had only one gene each (Fig. 2A). This uneven distribution indicates significant variation in the genomic abundance of *WAKL* genes in maize.

Gene duplication plays a crucial role in the expansion of gene families in plant evolution [32–35]. Among the analyzed *ZmWAKL* genes, 9 genes (16%) exhibited tandem duplication events within the genome. These tandem duplications occurred on chromosomes 2 and 8. By calculating Ka/Ks ratios, which represent the substitution ratios of non-synonymous to synonymous mutations, it was evident that most duplicated gene pairs underwent purifying selection during maize evolution (Table S3). The tandemly duplicated genes displayed a consistent number of introns, except for *ZmWAKL40* and *ZmWAKL41* Fig. 1C. No segmental duplication events were detected between different chromosomes. These findings indicate that tandem gene duplication events have contributed to the expansion of the *ZmWAKL* gene family in the maize genome.

A comparative syntenic analysis involving maize, barley, and rice was conducted to understand the evolutionary mechanisms underlying the *ZmWAKL* gene family (Fig. 2B). The analysis revealed that 21 *ZmWAKL* genes exhibited syntenic relationships with barley, while 28 genes showed syntenic relationships with rice. Multiple types of syntenic orthologous gene pairs were observed between barley and *ZmWAKL* genes, with certain maize genes corresponding to multiple barley genes and vice versa (Fig. 2B, Table S4). On the other hand, most of the 28 syntenic orthologous gene pairs between rice and maize exhibited a one-to-one syntenic relationship, indicating their origin from a common ancestor (Fig. 2B, Table S5). In some cases, one rice gene corresponded to multiple maize genes, and certain orthologous gene pairs were shared between maize and barley. These findings suggest the emergence of these orthologous gene pairs following the divergence of cereal crops.

### *Cis*-acting regulatory element of *ZmWAKLs*

The analysis of *ZmWAKL* genes' promoter region using the PlantCARE database revealed a wide range of *cis*-acting regulatory elements, indicating promoter diversity and the potential for intricate transcriptional regulation in these genes (Table S6). Conserved regulatory elements like the TATA-box and CAAT-box were identified across all *ZmWAKL* genes, highlighting their fundamental role in gene expression regulation. The presence of 10 *cis*-acting regulatory elements associated with hormone responses suggests the involvement of *ZmWAKL* genes in hormone-mediated processes (Table S7). These elements include auxin-responsive elements (AuxRR-core, TGA-element), abscisic acid-responsive element (ABRE), and gibberellin-responsive elements (GARE, P-box, TATC-box). Multiple hormone-responsive elements were found in each of the surveyed 58 *ZmWAKL* genes, with ABRE, GARE-motif, and TGACG-motif being the most prevalent, accounting for over 60% of the genes. Gibberellin responsiveness was of particular importance, as each of these 33 *ZmWAKL* genes contained at least one gibberellin-responsive motif, reinforcing the significance of gibberellin signaling in the regulation of these genes (Fig. S2 and Table S7). Additionally, certain *ZmWAKL* genes exhibited *cis*-acting regulatory elements associated with biotic and abiotic stress responses, suggesting their potential involvement in stress-related processes.

### The expression analysis of *WAKL* genes in maize seeds and GA-treated kernels at different development stages

**Fig. 3:**
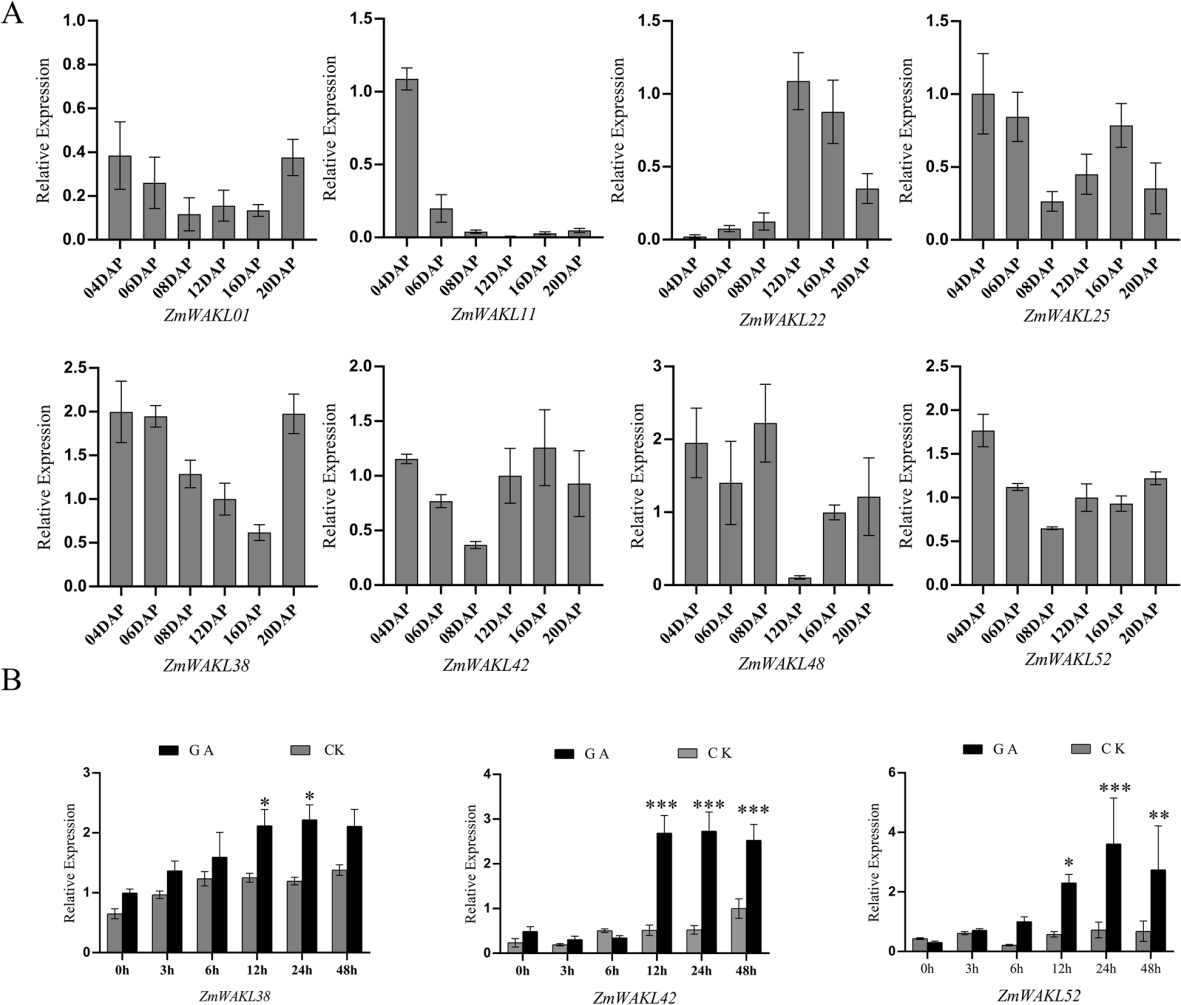
Expression analysis of 8 *ZmWAKL* genes. **A** RT-qPCR analysis of the 8 *WAKL* genes in maize seeds at various stages of development. The data is presented as the mean ± standard error (SE) of 6 replicates. DAP is the abbreviation for Day After Pollination. **B** Expression analysis of 3 *ZmWAKL* genes at different time points following GA treatment. The data is shown as the mean ± SE of at least five replicates. Statistical analysis was performed using a two-tailed paired *t*-test to determine the significance of the difference between GA-treated and control samples (*P < 0.05, **P < 0.01, ***P < 0.001)

The analysis of a high temporal-resolution transcriptome dataset provided valuable insights into the expression patterns of *ZmWAKL* genes during maize kernel development [36]. 14 *ZmWAKL* genes were identified during early seed development, according to previous studies [36]. However, in response to 50 mM GA treatment, only eight out of the 14 genes showed differential expression, indicating their involvement in the regulatory mechanisms of maize kernel development (Table S8) [24]. Analysis of the RNA-seq data, as indicated by ‘logFC(50 mm-GA-12 h/CK-12 h)’ in Table S8, referencing Lv et al.'s report [24], unveiled that all eight differentially expressed *ZmWAKL* genes demonstrated an upregulation in response to GA induction. Among them, *ZmWAKL38* and *ZmWAKL42* showed the most significant fold change in expression patterns. The validation through RT-qPCR analysis at multiple time points confirmed these expression profiles (Fig. 3A).

Further investigation focused on three selected genes, *ZmWAKL38*, *ZmWAKL42*, and *ZmWAKL52*, in maize kernels treated with 50 mM GA (Fig. 3B). The RT-qPCR analysis showed a consistent and significant upregulation of these genes after 12 h of GA treatment. While *ZmWAKL38* and *ZmWAKL42* displayed sustained elevated expression levels, *ZmWAKL52* reached its peak expression at 24 h, highlighting their positive transcriptional responsiveness to GA induction. In summary, the study provides insights into the dynamic expression patterns of *ZmWAKL* genes during maize kernel development.

### Co-expression of GA mediated TFs and *ZmWAKLs*

**Fig. 4:**
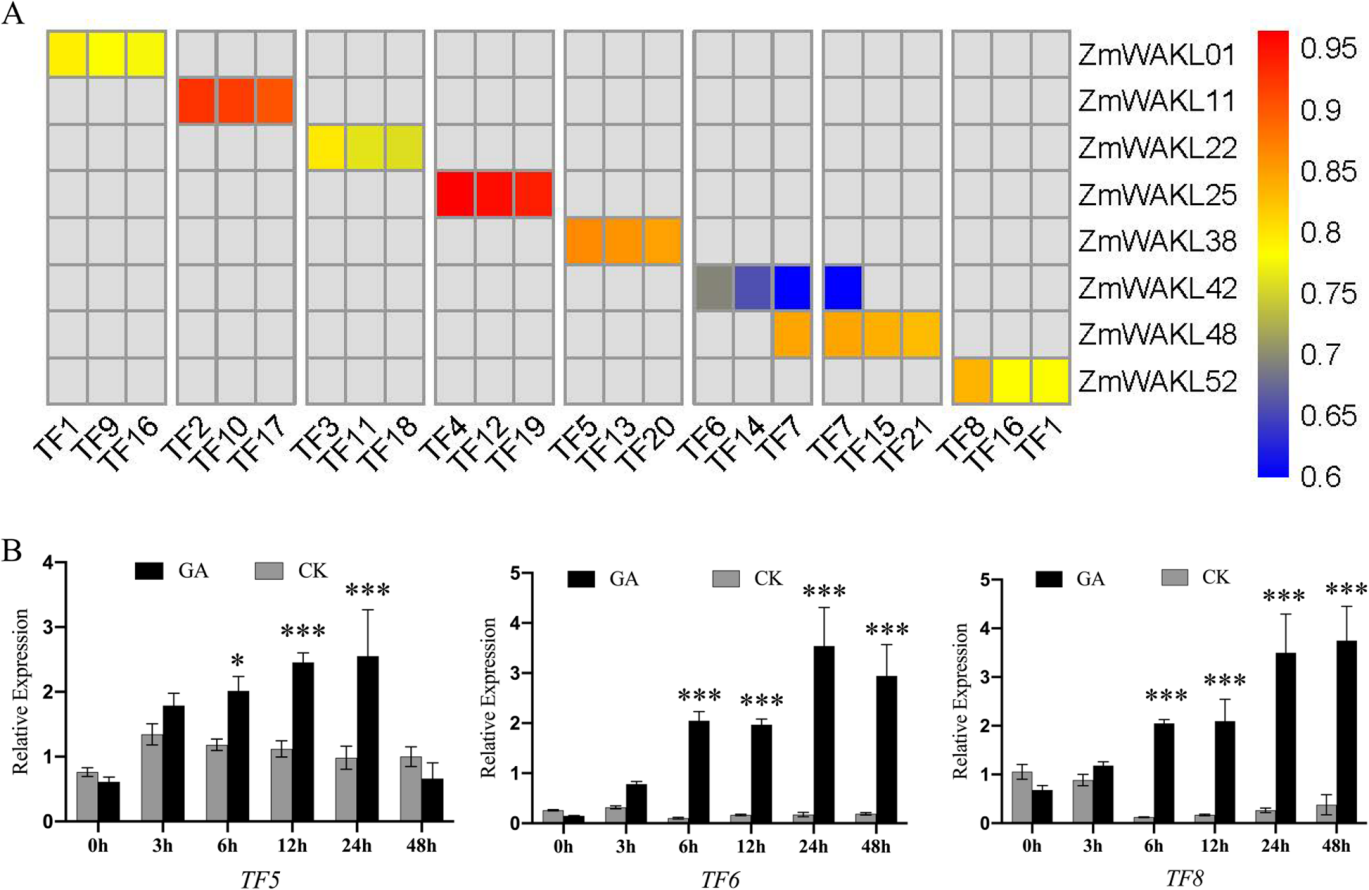
Co-expression analysis and expression analysis of TF5, TF6, TF8. **A** Hierarchical heatmap illustrating the co-expression patterns between 8 *ZmWAKL* genes and their respective differentially expressed TFs. Red indicates higher correlation coefficients, while blue represents lower correlation coefficients. TF1, TF7 and TF16 show high correlation coefficients with two *ZmWAKL*s, respectively. **B** Expression analysis of the 3 identified TFs following GA treatment. The data is presented as the mean ± standard error (SE) of five replicates. Statistical analysis was performed using a two-tailed paired t-test to determine the significance of the difference between GA-treated and control samples (*P < 0.05, **P < 0.01, ***P < 0.001)

Our investigation yielded evidence supporting the presence of GA-responsive elements in the promoters of *ZmWAKL38*, *ZmWAKL42*, and *ZmWAKL52* genes, with their expressions being positively regulated by GA treatment (Fig. 3 and Fig. 4B). These findings establish a direct link between GA signaling and the transcriptional regulation of these genes. Additionally, previous studies have indicated the involvement of *WAKL* genes in cell elongation and tissue morphology, emphasizing their significance in maize kernel development [7, 37].

To elucidate the transcriptional regulation of *ZmWAKL* genes by GA-responsive transcription factors (TFs), we performed a co-expression analysis [38, 39] using publicly available transcriptome data [24, 36]. This analysis revealed 21 potential TF candidates exhibiting co-expression patterns with the kernel highly expressed *ZmWAKL* genes (Fig. 4A). Specifically, TF5, TF6, and TF8 emerged as strong candidates for transcriptional regulation of *ZmWAKL38*, *ZmWAKL42*, and *ZmWAKL52*, respectively. Notably, TF5, belonging to the TALE family, has been shown to be inducible in response to wounds and sensitive to GA inactivation (Table S9). On the other hand, TF8, a member of the MYB family, is known for its responsiveness to phytohormones and its crucial role in various aspects of plant growth and development (Table S9).

To further investigate the responsiveness of TF5, TF6, and TF8 to GA induction, we conducted RT-qPCR analysis on maize kernels subjected to GA treatment at different time points (Fig. 4B). The results demonstrated a significant upregulation of these TFs following GA treatment, with peak transcript levels observed at different time intervals. TF5 exhibited a stabilization at 12 to 24 h, followed by a sharp decrease, while TF6 and TF8 maintained their elevated expression levels up to 24 h. The fold changes in transcript levels for these TFs ranged from 5 to 12, providing strong evidence for their responsiveness to GA induction. Consequently, the GA-induced expression of TF5, TF6, and TF8 likely contributes to the observed transcriptional alterations in *ZmWAKL* gene expression in GA-treated maize kernels. In summary, our study confirms that GA modulates the transcriptional regulation of *ZmWAKL* genes, and identifies potential TFs, such as TF5, TF6, and TF8, that are involved in this process.

### Identification of subcellular localization of *ZmWAKL*-encode protein and the candidate TFs

**Fig. 5:**
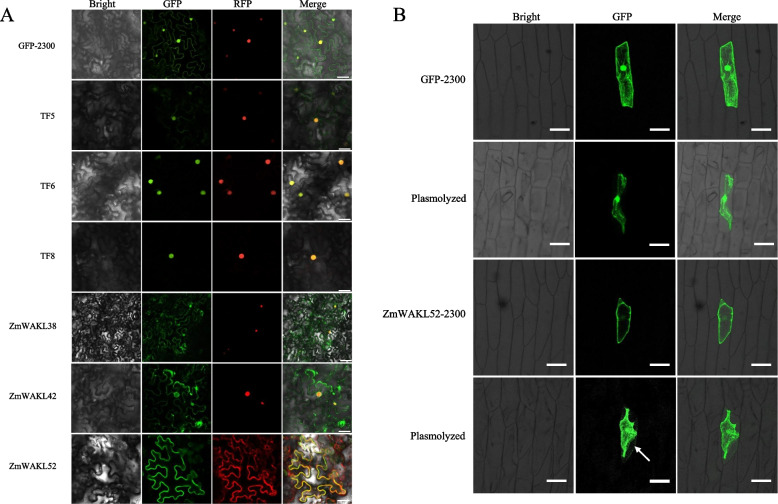
Analysis of subcellular localization for ZmWAKL proteins and TFs. **A** Subcellular localization analysis conducted in *N. benthamiana* leaves. NLS-RFP were used as nuclear marker and show red fluorescence signals. PIP2-cherry was exclusively employed as a cell membrane marker in conjunction with ZmWAKL52-GFP co-transformation in tobacco leaves, manifesting a distinctive red fluorescent signal. Scale bar = 25 µm. **B** Subcellular localization examination of *ZmWAKL52* in onion epidermal cells following plasmolysis. The arrow indicates GFP signals present in cell wall. Scale bar = 100 µm

The subcellular localization of *ZmWAKL*-encoding proteins, including *ZmWAKL38*, *ZmWAKL42*, and *ZmWAKL52*, as well as the candidate TFs, *TF5*, *TF6*, and *TF8*, was investigated in this study. To determine their localizations, the coding sequences of these proteins were fused to the GFP reporter gene, and their expressions were driven by the *CaMV 35S* promoter. Transient expression of these fusion constructs was carried out in *N. benthamiana* leaves, and after 24 h, confocal microscopy was used to examine the localization patterns within the cells. The results revealed distinct subcellular localizations for these proteins. TF6-GFP and TF8-GFP were found to predominantly localize in the nucleus. On the other hand, ZmWAKL38-GFP, ZmWAKL42-GFP, and TF5-GFP exhibited fluorescence signals in both the cytoplasm and nucleus. This suggests that TF6 and TF8 likely function as nuclear proteins, while TF5 may play a role in both the cytoplasm and nucleus (Fig. 5A).

According to the results depicted in Fig. 5A and Fig. S3, ZmWAKL52-GFP exhibited fluorescence patterns in the plasma membrane. Furthermore, a closer investigation was carried out for *ZmWAKL52* to understand its binding properties to the cell membrane and cell wall. Onion epidermal cells were transformed with ZmWAKL52-GFP and subjected to plasmolysis. The plasmolyzed cells showed GFP signals from ZmWAKL52-GFP in both the cytoplasm and cell wall, indicating its localization in the cytoplasm and its association with the cell wall (Fig. 5B). In summary, the study identified the subcellular localizations of *ZmWAKL*-encoding proteins and candidate TFs. TF6 and TF8 primarily localized to the nucleus, while ZmWAKL38, ZmWAKL42, and TF5 exhibited fluorescence signals in both the cytoplasm and nucleus. Additionally, ZmWAKL52 was found to associate with the plasma membrane and cell wall.

### GA induced TFs regulated the expression of *ZmWAKLs*

**Fig. 6:**
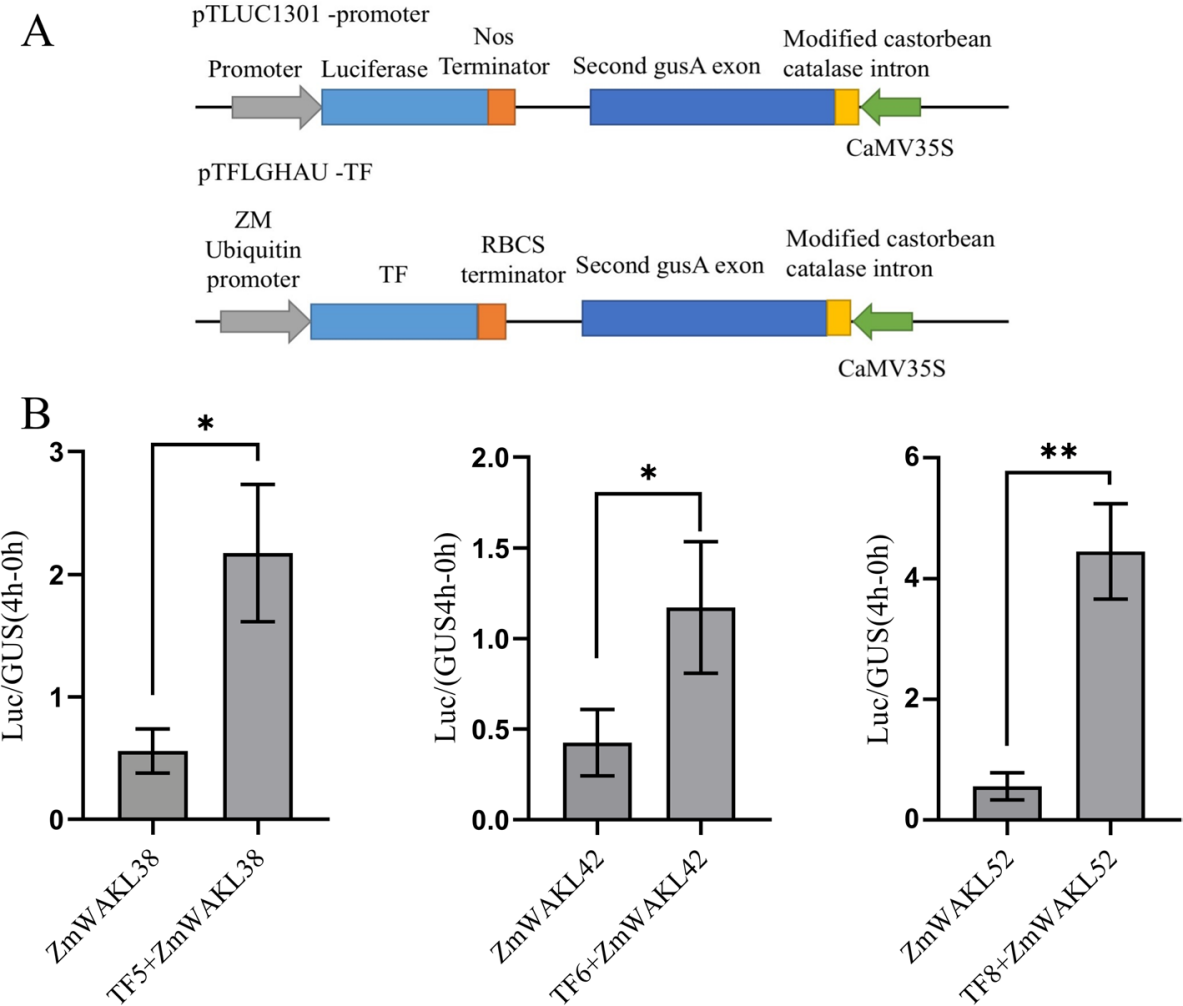
Analysis of TF-promoter interaction using a transient assay in *N. benthamiana* leaves. **A** Schematic representation of the plasmid constructs employed in the experiment. **B** Co-transformation of pTLUC1301-promoter and pTFLGHAU-TF plasmids in *N. benthamiana* leaves. LUC activity was standardized to GUS activity in each individual transformation. The data represent the means ± SD of 6 replicates. The statistical significance of differences between -TF and + TF samples was assessed using a two-tailed paired *t*-test (*P < 0.05, **P < 0.01)

The objective of this study was to investigate the regulatory influence of GA-induced TFs, specifically TF5, TF6, and TF8, on the expression of *ZmWAKL* genes. To evaluate their impact on transcriptional regulation, a transient promoter activity assay was conducted in *N. benthamiana* plant leaves. For this purpose, separate plasmids were constructed, encompassing the promoter regions of *ZmWAKL38*, *ZmWAKL42*, and *ZmWAKL52*, along with the coding sequences of TF5, TF6, and TF8.These plasmids were individually co-transformed into *N. benthamiana* leaves, enabling the measurement of reporter gene activities. To ensure accurate comparisons, β-Glucuronidase (GUS) activity of the second GUS exon in the pTLUC1301 was used as an internal control to normalize the transformation efficiency. The plasmid structure used in the transcriptional activity assay is illustrated in Fig. 6A. The results of the assay demonstrated that TF5, TF6, and TF8 significantly augmented the transcriptional activities of the *ZmWAKL38*, *ZmWAKL42*, and *ZmWAKL52* promoters, respectively. Figure 6B illustrates the observed elevation in luciferase (LUC) activity compared to GUS activity. Notably, the TF8 + ZmWAKL52 combination exhibited the highest LUC/GUS activity ratio, followed by TF5 + ZmWAKL38.In conclusion, these findings provide evidence that GA-induced TFs, including TF5, TF6, and TF8, possess the ability to modulate the transcriptional expression of *ZmWAKL38*, *ZmWAKL42*, and *ZmWAKL52* genes in maize.

## Discussion

WAK/WAKL proteins are important receptor-like kinases that play a crucial role in perceiving signals from the extracellular environment and facilitating cellular responses in plants. Their involvement spans various processes, such as disease resistance, phytohormone signaling, and stress responses, which enable plants to adapt to changing environmental conditions [12, 27, 28]. It is noteworthy that *WAK*-mediated cell expansion has been associated with pathogen defense, exhibiting diverse molecular mechanisms. In maize, the *WAK/WAKL* family significantly contributes to disease resistance. For instance, *ZmWAKL19* (also known as *ZmWAK*) plays a pivotal role in inhibiting the growth of the fungal pathogen *Sporisorium reilianum*, causing head smut disease [40]. This defense mechanism relies on promoting cell growth and inducing apoptosis-like programmed cell death (AL-PCD) to restrict the hyphal growth of the pathogen, enhancing resistance against head smut disease. Another member of the *WAK/WAKL* family in maize, *ZmWAKL53*, is involved in defense against the hemibiotrophic fungal pathogen *Exserohilum turcicum* [13]. It localizes to the plasma membrane and acts to reduce pathogen penetration into host tissues. This defense response is accompanied by changes in benzoxazinoid metabolites and downstream biochemical fluxes starting from indole-3-glycerol phosphate.

Furthermore, WAK/WAKL proteins, in addition to their role in disease resistance, have broader impacts on various cellular processes such as cell expansion and morphogenesis. For example, silencing *OsWAK1* in rice results in reduced cell size and a dwarf phenotype [6], while downregulating *WAK4* in *Arabidopsis* inhibits cell elongation [7], hinders root development, leading to dwarfism and sterility. Given the pivotal role of *WAK/WAKL* in regulating cell growth processes critical for normal plant development, it is conceivable that they are subject to the influence of various plant hormones [41–43], participate in specific points of cell wall synthesis, and may even involve unknown regulatory mechanisms. Subsequent research efforts, possibly employing multi-omics approaches, could focus on analyzing *WAK/WAKL* genetic materials with distinct phenotypic differences to uncover potential regulatory molecular mechanisms.

During the investigation of *ZmWAKL* gene regulation in maize, it was discovered that their promoters contain hormone-responsive elements, particularly GA-responsive elements. GA positively regulates *ZmWAKL38*, *ZmWAKL42*, and *ZmWAKL52*, with transcriptional regulation mediated by GA-responsive transcription factors like TF5, TF6, and TF8 during seed development. Considering the subcellular localization of *ZmWAKL52* in the cell wall and plasma membranes, GA partially interacts with the *WAKL* family, which is known for promoting cell differentiation and expansion [44, 45]. Despite the revelation of GA's involvement in mediating *ZmWAKL* gene expression, the detailed molecular mechanisms and their impact on maize development remain unknown. Further research is needed to ascertain the functional importance of *ZmWAKL* genes, their roles in cell wall interactions, and their broader impact on maize development and yield. Additionally, it is crucial to investigate whether the subcellular localization of *ZmWAKL* genes changes after GA treatment, elucidate the molecular mechanisms underlying GA-mediated transcriptional regulation of *ZmWAKL*, explore the specific types of GA involved in *ZmWAKL* transcriptional expression, identify potential GA receptors, and unravel their interactions with GA-responsive TFs. Moreover, verifying whether *ZmWAKL* is regulated by other plant hormones such as auxin, ABA, SA, among others, is essential to clarify the specificity of GA regulation on *ZmWAKL*. Subsequent studies can indeed focus more on these aspects for providing deeper insights and facilitating the formulation of hypotheses based on the findings.

Additionally, the potential role of *ZmWAKL* genes in grain development and yield deserves mention. According to the subcellular localization experiments in this study, *ZmWAKL52* is localized to the cell wall, and its expression is positively regulated by GA-mediated transcription. Given the commonality of the *WAKL* family in promoting cell wall synthesis, we hypothesize that *ZmWAKL52*, under the control of the GA-induced transcription factor TF8, may enhance cell wall synthesis, leading to increased cell volume, thereby augmenting grain size, and enhancing maize yield. In subsequent research, we recommend conducting experiments to specifically investigate changes in cell wall composition in response to GA-induced cell wall synthesis related to *ZmWAKL52*. It is known that *WAK* gene family members can enhance cell wall elasticity by promoting pectin synthesis, consequently increasing cell size. Additionally, an exploration of potential interaction factors facilitated by GA-mediated TF8 is suggested to elucidate the mechanistic relationship between interaction factors and *ZmWAKL52* in cell wall synthesis, further explicating the molecular mechanism by which *ZmWAKL52* promotes cell wall synthesis. Understanding the influence of *ZmWAKL* genes on grain development and yield will provide valuable insights for enhancing crop productivity and implementing effective strategies for maize improvement.

## Conclusion

The discovery of 58 *WAKL* members in maize highlights their potential significance in plant development and cell wall expansion. Importantly, the functional conservation of these *ZmWAKL* genes has been successfully demonstrated. This study specifically focused on three genes, namely *ZmWAKL38*, *ZmWAKL42*, and *ZmWAKL52*, and their promoters were analyzed, revealing the presence of GA-responsive elements, implying their regulation by GA. Furthermore, we have determined that the expressions of these three genes are transcriptionally controlled by GA-responsive transcription factors, namely TF5, TF6, and TF8. These findings shed light on the regulatory mechanisms of the maize *WAKL* family genes and lay a solid foundation for future investigations into their functional roles in maize development. Understanding the interplay between GA and the transcriptional regulation of *ZmWAKL* genes is essential for unraveling the intricate pathways underlying plant growth and adaptation. The insights gained from this study contribute to a broader understanding of the intricate molecular processes governing maize development and provide a starting point for further in-depth exploration of the maize *WAKL* family genes.

## Materials and Methods

### Optimization and Identification of Maize-specific *WAKL* Genes

To optimize the identification of maize *WAKL* genes, protein sequences from the maize genome B73 were obtained from the MaizeGDB database (https://www.maizegdb.org) [46]. An initial HMM profile was established using the Pfam protein family database (http://pfam.xfam.org), incorporating three domains: EGF_Ca domain (PF07645), GUB_WAKL domain (PF13947), and protein tyrosine kinase domain (PF07714). The HMMER 3.3.2 software (http://hmmer.org) was utilized with an e-value cut-off of 0.001 to search for maize *WAKL* genes within the protein sequences [47]. The search targeted genes containing the EGF_Ca or GUB_WAKL domains, with the protein tyrosine kinase domain as an optional component [48]. Confirmation of the presence of WAKL core sequences was performed using the PFAM and SMART programs. Only genes possessing the EGF_Ca or GUB_WAKL domains, with the protein tyrosine kinase domain being optional, were selected. The selected genes underwent alignment using the ClustalW software to identify similarities and conserved regions. Based on this alignment, a maize-specific WAKL HMM profile capturing unique characteristics and variations was constructed. Using the optimized maize-specific WAKL HMM profile, the HMMER software was employed to identify *ZmWAKL* family genes. The same steps were followed to identify genes with the required domains.

### Phylogenetic relationship, conserved motifs, and gene structure analysis of *ZmWAKL*

The protein sequences of *ZmWAKL* genes were aligned using the MUSCLE algorithm. A phylogenetic tree was constructed using the neighbor-joining method in Mega7 [49]. The MEME program (http://meme-suite.org) was utilized to predict conserved motifs within ZmWAKL proteins [50]. The motifs were annotated using CDD, SMART, and InterPro software. To visualize the gene structure of *ZmWAKL*, including intron and exon information, the TBtools software (https://github.com/CJ-Chen/TBtools) was employed, enabling graphical representations of gene structures and enhanced understanding of exon–intron organization and other structural features.

### Chromosomal localization and gene duplication analysis

Chromosomal positions of *ZmWAKL* genes were obtained from the MaizeGDB database, specifically utilizing the B73 AGPv4 genome assembly. The Mapgene2chrom software (http://mg2c.iask.in/mg2c_v2.0/) was used to visually represent the chromosomal distribution of the *ZmWAKL* genes [51]. Gene duplication events of *ZmWAKL* in maize were explored based on alignment coverage of > 60% of the longer gene, alignment identity of > 60% in the aligned region, and genomic location. Tandem duplicated genes were identified when located within the same chromosomal fragment of less than 100 kb, separated by five or fewer genes. Segmental duplication events involving *ZmWAKL* genes were identified through the Plant Genome Duplication Database (http://chibba.agtec.uga.edu/duplication/) [52].

### *Cis*-acting element analysis and expression pattern of *ZmWAKLs*

The 2000 bp upstream sequences of *ZmWAKL* genes obtained from the B73_RefGen_v4 genome assembly were analyzed for *cis*-acting elements using the PlantCare (http://bioinformatics.psb.ugent.be/webtools/plantcare/html/) [53]. Expression profiles of *ZmWAKL* genes and transcription factors during the early stages of maize seed development were retrieved from the transcriptome results [36]. These profiles provided insights into the temporal and spatial expression patterns of *ZmWAKL* genes.

### Plant materials

A comprehensive investigation was conducted during the 2021 growing season at the research field of Sichuan Agricultural University in Chongzhou, China, with the aim of understanding the cultivation and management of maize inbred line B73. The study involved the collection of seeds at different developmental stages, including 6 DAP, 4 DAP, 6 DAP, 8 DAP, 12 DAP, 16 DAP, and 20 DAP, followed by mRNA extraction. Additionally, genomic DNA was extracted from maize leaves at the 3rd leaf stage. To explore gene expression and developmental changes in maize, kernels from independent ears were harvested at 10 DAP and treated with 50 mM GA for multiple treatment time. Samples were collected at various time points (0 h, 3 h, 6 h, 12 h, 24 h, and 48 h), with untreated ears at the same developmental stage serving as control samples. To preserve the collected kernels, they were promptly frozen in liquid nitrogen and stored at -80˚C for subsequent analysis, enabling a comprehensive understanding of gene expression dynamics and developmental processes in maize.

### Gene cloning and plasmid construction

To acquire the necessary genetic materials, we utilized cDNA isolated from a combination of maize kernels aged 4–20 days after pollination (DAP) for cloning *ZmWAKL* genes and candidate TF genes. For cloning *ZmWAKL* gene promoters, we employed genomic DNA. Amplification of all targeted genes and promoters was achieved through the utilization of the Phanta Max Master Mix (Vazyme, P511-01), followed by their cloning into multiple plasmids using the ClonExpress II One Step Cloning Kit (Vazyme, C112-01) for subsequent sequencing and experimental procedures.

In this study, we conducted a transcriptional activation assay using the pTLUC1301 and pTFLGHAU vectors. The pTLUC1301 vector, a modified variant of pCAMBIA1301, served as the fundamental backbone and contained two reporter genes, namely luciferase (LUC) and β-glucuronidase (GUS). In pTLUC1301, the *CaMV35* promoter and *HygR* gene from pCAMBIA1301 were replaced by the *LUC* gene. The *GUS* gene, driven by the *CaMV35* promoter in pTLUC1301, served as a reference gene for measuring the efficiency of tobacco cell transformation in transient expression assays. The activity of β-glucuronidase was used as an internal control to standardize the transformation efficiency. The gene promoter of interest was inserted upstream of the LUC gene in the pTLUC1301 plasmid.

Additionally, the coding sequences of the TFs were inserted into the pTFLGHAU vector, downstream of the RBCS terminator. The maize Ubiquitin promoter drove the expression of TFs in this vector. For the subcellular localization analysis, we employed the p2300-eGFP plasmid. To achieve this, we amplified the CDS of *ZmWAKL* and *TF*s, incorporating K*pn*I and X*ba*I restriction sequences, and subsequently cloned them into p2300-eGFP. The resulting recombinant plasmids were named pWAKL-2300 s and pTF-2300 s. PIP2-cherry plasmid was used as plasma membrane marker and plasmid construction was according to the previous reports [54]. The details of NLS-RFP plasmid construction were reported [55]. Specific primers, listed in Table S10, were employed for gene/promoter cloning and plasmid construction.

### Subcellular localization analysis and transcriptional activation assay

To perform the subcellular localization and transcriptional activation assays, we transferred the plasmids into *Agrobacterium tumefaciens* strain GV3101. NLS-RFP served as a nuclear marker, as previously described [55, 56]. For the subcellular localization assay, we introduced *A. tumefaciens* strains carrying the expression plasmids, along with the NLS-RFP plasmid, into the abaxial air space of 2 to 4-week-old *Nicotiana benthamiana* leaves via infiltration. Following a 24-h incubation period, we examined the fluorescence signals in the epidermal cell layers near the infiltration sites using a confocal microscope from Leica Microsystems, Germany. To examine the localization of *ZmWAKL52* within the plasma membrane, PIP2-cherry was co-transformed exclusively with pZmWAKL52-2300 into tobacco leaves, serving as a plasma membrane marker. For the *A. tumefaciens*-mediated transformation of onion epidermal cells, we followed a previously published method [57]. Subsequently, we incubated the transformed onion epidermal cells in the dark at 28 °C for 24 h. To analyze GFP expression in plasmolyzed cells, we treated the transformed onion epidermal cells with a 0.8 M mannitol solution for 15 min before observing GFP expression [41].

In the transcriptional activation assay, we utilized *Nicotiana benthamiana* leaves as the experimental system. We followed the *A. tumefaciens* transformation and infiltration protocols described in Zhang's study, with slight modifications tailored to our experiment [41]. After a 48-h treatment period, we harvested the infiltrated *Nicotiana benthamiana* leaves and immediately froze them in liquid nitrogen. The frozen samples were then ground, and the resulting material was used to determine the ratio of LUC activity to GUS (β-glucuronidase) activity. The methodology for determining the LUC activity/GUS activity ratio was according to the work of Hu et al. [58]. To normalize the transformation efficiency, we employed the activity of *CaMV35S*-driven β-*glucuronidase* in pTLUC1301 as the internal control [59]. Statistical analysis involved the calculation of average values and variability among replicates using Excel 2016. We conducted a two-tailed paired *t*-test to assess the statistical significance of observed distinctions. A threshold of *P < 0.05 was adopted to denote statistical significance, while **P < 0.01 was employed to signify a higher level of significance.

### Co-expression analysis and RT-PCR assay

In accordance with the methods reported by Chen et al. [39], a co-expression analysis was conducted to identify the correlation coefficients between GA up-regulated TFs and the 8 kernel-high-expressed *ZmWAKL*. The expression compartment in kernels of these *ZmWAKL*s were based on eFP Browsers for tissue-specific expression determined by Hoopes GM et al. [60]. The GA-induced kernel RNA-sequencing results and high temporal transcriptome data of kernel were employed to derive correlation coefficients for TFs and *ZmWAKL* [24, 36]. Among them, the top three TFs exhibiting significant correlation coefficients were designated as prospective GA-mediated TFs for the eight *ZmWAKL* genes (Table S9). To assess gene expression, we extracted total RNA from maize inbred line B73 seeds at different developmental stages (4 DAP, 6 DAP, 8 DAP, 12 DAP, 16 DAP, 20 DAP) and from protoplasts. RNA isolation was carried out using the TRIzol reagent from Invitrogen following the manufacturer's instructions. The first-strand cDNA synthesis was performed using the PrimeScriptRT reagent Kit with gDNA Eraser from TaKaRa, starting from 1.5 μg of total RNA. The maize Actin gene was amplified using the ACTINF and ACTINR primers as an internal control. To analyze the expression of the eight *ZmWAKL*s in maize seeds at different developmental stages, we employed RT-qPCR. Additionally, we examined the expression of *ZmWAKL*s and TFs in GA-treated kernels at various time points (0 h, 3 h, 6 h, 12 h, 24 h, and 48 h) using RT-qPCR. For all PCR experiments, we used the Bio-Rad CFX96 real-time PCR detection system with cycle conditions provided in the instructions of the SYBR PrimeScript RT-PCR Kit from Takara. The specific primer sequences used can be found in Table S10. The statistical methods used were as described previously.

### Supplementary Information

**Additional file 1: Fig. S1.**

**Additional file 2: Fig. S2. **

**Additional file 3: Fig. S3. **

**Additional file 4: Table S1.1. **WAKL gene and protein sequence information.** Table S1.2. **Ten predicted conserve motifs of ZmWAKLs.** Table S1.3. **Distribution of predicted EGF-domains among ZmWAKL.

**Additional file 5: Table S2. **Basic information of ZmWAKL proteins

**Additional file 6: Table S3. **Non-synonymous substitution (Ka), rate of synonymous substitutions (Ks) and Ka/Ks of *ZmWAKL* genes

**Additional file 7: Table S4. **21 *ZmWAKL* genes showed syntenic relationship with those in barley

**Additional file 8: Table S5. **28 *ZmWAKL* genes showed syntenic relationship with those in rice

**Additional file 9: Table S6. **Promoter motif prediction of 58 *ZmWAKL*.

**Additional file 10: Table S7. **10 Phytohormone related *cis*-acting elements in the promoter of *ZmWAKLs*

**Additional file 11: Table S8. **14 *ZmWAKLs* expression information of multiple seed development stage and log values of 10 DAP seeds after 50 mM GA treatment

**Additional file 12: Table S9. **Gene ID and annoantion  of the TFs from Co-expression analysis

**Additional file 13: Table S10. **Primers used in this paper

## Data Availability

The data and materials pertaining to the current study have been incorporated in the article or made available as supplementary information. Any additional inquiries regarding the study may be directed to the corresponding authors. The datasets generated during the study are deposited in publicly accessible repositories such as GenBank (http://www.ncbi.nlm.nih.gov/Genbank) and MaizeGDB (http://www.maizegdb.org), with transparent and unrestricted public access. The entirety of the data generated or analyzed during the course of the study is included in both the article and its supplementary information files. The assigned accession numbers for these datasets are as follows: Zm00001d030942, Zm00001d031338, Zm00001d031351, Zm00001d031366, Zm00001d031372, Zm00001d031395, Zm00001d031407, Zm00001d031466, Zm00001d032356, Zm00001d002039, Zm00001d002446, Zm00001d002447, Zm00001d002979, Zm00001d003019, Zm00001d003021, Zm00001d003023, Zm00001d003069, Zm00001d003070, Zm00001d007745, Zm00001d039920, Zm00001d039926, Zm00001d039931, Zm00001d040094, Zm00001d041873, Zm00001d043800, Zm00001d048631, Zm00001d050164, Zm00001d053079, Zm00001d054062, Zm00001d017264, Zm00001d018431, Zm00001d035479, Zm00001d036261, Zm00001d036263, Zm00001d036999, Zm00001d037635, Zm00001d038915, Zm00001d020955, Zm00001d008457, Zm00001d008458, Zm00001d008462, Zm00001d008467, Zm00001d008468, Zm00001d008477, Zm00001d008485, Zm00001d008488, Zm00001d008489, Zm00001d008581, Zm00001d008585, Zm00001d008586, Zm00001d010442, Zm00001d011625, Zm00001d011629, Zm00001d045190, Zm00001d023563, Zm00001d024961, Zm00001d024962, Zm00001d026004, Zm00001d050893, Zm00001d017409,Zm00001d031790,Zm00001d052357, Zm00001d027802,Zm00001d006551, Zm00001d036768, Zm00001d041958, Zm00001d027852, Zm00001d017112, Zm00001d032024, Zm00001d039496,Zm00001d021946, Zm00001d013074, Zm00001d011969, Zm00001d045398, Zm00001d008323, Zm00001d012527, Zm00001d049079, Zm00001d038189, Zm00001d053819.

## Abbreviations

WAK: Wall Associated Kinase
RLK: Receptor-Like Kinase
EGF: Epidermal Growth Factor
WAKL: Wall Associated Kinase Like
ABA: Abscisic Acid
MeJA: Methyl Jasmonate
SA: Salicylic Acid
GA: Gibberellic Acid
TF: Transcription Factor
GUS: β-Glucuronidase
LUC: Luciferase
AL-PCD: Apoptosis-Like Programmed Cell Death
RT-qPCR: Real-Time Quantitative PCR
DAP: Days After Pollination

## References

[1] Somerville C, Bauer S, Brininstool G (2004). Toward a systems approach to understanding plant cell walls. Science.

[2] Humphrey TV, Bonetta DT, Goring DR (2007). Sentinels at the wall: cell wall receptors and sensors. New Phytol.

[3] Wagner TA, Kohorn BD (2001). Wall-associated kinases are expressed throughout plant development and are required for cell expansion. Plant Cell.

[4] Kaur R, Singh K, Singh J (2013). A root-specific wall-associated kinase gene, *HvWAK1*, regulates root growth and is highly divergent in barley and other cereals. Funct Integr Genomics.

[5] Sivaguru M, Ezaki B, He ZH (2003). Aluminum-induced gene expression and protein localization of a cell wall-associated receptor kinase in *Arabidopsis*. Plant Physiol.

[6] Kanneganti V, Gupta AK (2011). RNAi mediated silencing of a wall associated kinase, *OsiWAK1* in *Oryza sativa* results in impaired root development and sterility due to anther indehiscence: Wall Associated Kinases from *Oryza sativa*. Physiol Mol Biol Plants.

[7] Lally D, Ingmire P, Tong HY (2001). Antisense expression of a cell wall–associated protein kinase, *WAK4*, inhibits cell elongation and alters morphology. Plant Cell.

[8] Kohorn BD (2016). Cell wall-associated kinases and pectin perception. J Exp Bot..

[9] Zhang Z, Ma W, Ren Z (2021). Characterization and expression analysis of *wall-associated kinase* (*WAK*) and *WAK-like* family in cotton. Int J Biol Macromolecules.

[10] Tripathi RK, Aguirre JA, Singh J (2021). Genome-wide analysis of *wall associated kinase* (*WAK*) gene family in barley. Genomics.

[11] Zuo C, Liu Y, Guo Z (2019). Genome-wide annotation and expression responses to biotic stresses of the *WALL-ASSOCIATED KINASE-RECEPTOR-LIKE KINASE* (*WAK-RLK*) gene family in Apple (*Malus domestica*). Eur J Plant Pathol.

[12] Zuo W, Chao Q, Zhang N (2015). A maize wall-associated kinase confers quantitative resistance to head smut. Nat Genet.

[13] Yang P, Praz C, Li B (2019). Fungal resistance mediated by maize wall-associated kinase ZmWAK-RLK1 correlates with reduced benzoxazinoid content. New Phytol.

[14] Yang P, Scheuermann D, Kessel B (2021). Alleles of a *wall-associated kinase* gene account for three of the major northern corn leaf blight resistance loci in maize. Plant J.

[15] Ford BA, Foo E, Sharwood R (2018). *Rht18* semidwarfism in wheat is due to increased *GA 2-oxidaseA9* expression and reduced GA content. Plant Physiol.

[16] Hou H, Zheng X, Zhang H (2017). Histone deacetylase is required for GA-induced programmed cell death in maize aleurone layers. Plant Physiol.

[17] Liu S, Zhang Y, Feng Q (2018). Tomato AUXIN RESPONSE FACTOR 5 regulates fruit set and development via the mediation of auxin and gibberellin signaling. Sci Rep.

[18] Depuydt S, Hardtke CS (2011). Hormone signalling crosstalk in plant growth regulation. Curr Biol.

[19] Rizza A, Jones AM (2019). The makings of a gradient: spatiotemporal distribution of gibberellins in plant development. Curr Opin Plant Biol.

[20] Majda M, Robert S (2018). The role of auxin in cell wall expansion. Int J Mol Sci.

[21] Camoni L, Visconti S, Aducci P (2018). 14-3-3 proteins in plant hormone signaling: doing several things at once. Front Plant Sci.

[22] Bai WQ, Xiao YH, Zhao J (2014). Gibberellin overproduction promotes sucrose synthase expression and secondary cell wall deposition in cotton fibers. PLoS ONE.

[23] Rebetzke GJ, Richards RA (2000). Gibberellic acid-sensitive dwarfing genes reduce plant height to increase kernel number and grain yield of wheat. Aust J Agric Res.

[24] Lv H, Li X, Li H (2021). Gibberellin induced transcription factor *bZIP53* regulates *CesA1* expression in maize kernels. PLoS ONE.

[25] Li Y, Ma S, Zhao Q (2021). *ZmGRAS11*, transactivated by Opaque2, positively regulates kernel size in maize. J Integr Plant Biol.

[26] Mahender S, Narendra K, Tomar S (2018). Effect of gibberllic acid on growth, yield and economics of maize *(Zea mays* L.). J Anim Sci.

[27] Yu H, Zhang W, Kang Y (2022). Genome-wide identification and expression analysis of wall-associated kinase (WAK) gene family in potato (Solanum tuberosum L.). Plant Biotechnol Rep.

[28] Dou L, Li Z, Shen Q (2021). Genome-wide characterization of the *WAK* gene family and expression analysis under plant hormone treatment in cotton. BMC Genomics.

[29] Zhang S, Chen C, Li L (2005). Evolutionary expansion, gene structure, and expression of the rice *wall-associated kinase* gene family. Plant Physiol.

[30] Nakhamchik A, Zhao Z, Provart NJ (2004). A comprehensive expression analysis of the *Arabidopsis* proline-rich extensin-like receptor kinase gene family using bioinformatic and experimental approaches. Plant Cell Physiol.

[31] Li M, Ma J, Liu H (2022). Identification and characterization of *wall-associated kinase (WAK)* and *WAK-like (WAKL)* gene family in *Juglans regia* and its wild related species *Juglans mandshurica*. Genes.

[32] Flagel LE, Wendel JF (2009). Gene duplication and evolutionary novelty in plants. New Phytol.

[33] Cannon SB, Mitra A, Baumgarten A (2004). The roles of segmental and tandem gene duplication in the evolution of large gene families in *Arabidopsis thaliana*. BMC Plant Biol.

[34] Kent WJ, Baertsch R, Hinrichs A (2003). Evolution's cauldron: duplication, deletion, and rearrangement in the mouse and human genomes. Proc Natl Acad Sci.

[35] Mehan MR, Freimer NB, Ophoff RA (2004). A genome-wide survey of segmental duplications that mediate common human genetic variation of chromosomal architecture. Hum Genomics.

[36] Yi F, Gu W, Chen J (2019). High temporal-resolution transcriptome landscape of early maize seed development. Plant Cell.

[37] Yue ZL, Liu N, Deng ZP (2022). The receptor kinase OsWAK11 monitors cell wall pectin changes to fine-tune brassinosteroid signaling and regulate cell elongation in rice. Curr Biol.

[38] Fu FF, Xue HW (2010). Coexpression analysis identifies Rice Starch Regulator 1, a rice AP2/EREBP family transcription factor, as a novel rice starch biosynthesis regulator. Plant Physiol.

[39] Chen J, Yi Q, Cao Y (2016). ZmbZIP91 regulates expression of starch synthesis-related genes by binding to ACTCAT elements in their promoters. J Exp Bot.

[40] Zhang N, Zhang B, Zuo W (2017). Cytological and molecular characterization of *ZmWAK*-mediated head-smut resistance in maize. Mol Plant Microbe Interact.

[41] Wang H, Niu H, Liang M (2019). A wall-associated kinase gene *CaWAKL20* from pepper negatively modulates plant thermotolerance by reducing the expression of ABA-responsive genes. Front Plant Sci.

[42] Yang K, Qi L, Zhang Z (2014). Isolation and characterization of a novel wall-associated kinase gene *TaWAK5* in wheat (*Triticum aestivum*). The Crop Journal.

[43] Yang J, Xie M, Wang X (2021). Identification of cell wall-associated kinases as important regulators involved in *Gossypium hirsutum* resistance to *Verticillium dahliae*. BMC Plant Biol.

[44] Ueguchi-Tanaka M, Nakajima M, Motoyuki A (2007). Gibberellin receptor and its role in gibberellin signaling in plants. Annu Rev Plant Biol.

[45] Hernández-García J, Briones-Moreno A, Blázquez MA (2021). Origin and evolution of gibberellin signaling and metabolism in plants. Semin Cell Dev Biol.

[46] Portwood JL, Woodhouse MR, Cannon EK (2019). MaizeGDB 2018: the maize multi-genome genetics and genomics database. Nucleic Acids Res.

[47] Mistry J, Finn RD, Eddy SR (2013). Challenges in homology search: HMMER3 and convergent evolution of coiled-coil regions. Nucleic Acids Res.

[48] Letunic I, Khedkar S, Bork P (2021). SMART: recent updates, new developments and status in 2020. Nucleic Acids Res.

[49] Kumar S, Stecher G, Tamura K (2016). MEGA7: molecular evolutionary genetics analysis version 7.0 for bigger datasets. Mol Biol Evol.

[50] Bailey TL, Elkan C (1994). Fitting a mixture model by expectation maximization to discover motifs in bipolymers. Proc Int Conf Intell Syst Mol Biol.

[51] Chao JT, Kong YT, Wang Q (2015). MapGene2Chrom, a tool to draw gene physical map based on Perl and SVG languages. Hereditas.

[52] Lee TH, Kim J, Robertson JS (2017). Plant genome duplication database. Methods Mol Biol.

[53] Lescot M, Déhais P, Thijs G (2002). PlantCARE, a database of plant *cis*-acting regulatory elements and a portal to tools for *in silico* analysis of promoter sequences. Nucleic Acids Res.

[54] Simon MLA, Platre MP, Assil S (2014). A multi-colour/multi-affinity marker set to visualize phosphoinositide dynamics in *Arabidopsis*. Plant J.

[55] Huang YY, Shi Y, Lei Y (2014). Functional identification of multiple nucleocytoplasmic trafficking signals in the broad-spectrum resistance protein RPW8.2. Planta.

[56] Zhao JH, Huang YY, Wang H (2023). Golovinomyces cichoracearum effector-associated nuclear localization of RPW8. 2 amplifies its expression to boost immunity in Arabidopsis. New Phytol.

[57] Sun W, Cao Z, Li Y (2007). A simple and effective method for protein subcellular localization using *Agrobacterium*-mediated transformation of onion epidermal cells. Biologia.

[58] Hu YF, Li YP, Zhang J (2012). Binding of ABI4 to a CACCG motif mediates the ABA-induced expression of the ZmSSI gene in maize (Zea mays L.) endosperm. J Exp Bot.

[59] Li Y, Yu G, Lv Y (2018). Combinatorial interaction of two adjacent *cis*-active promoter regions mediates the synergistic induction of *Bt2* gene by sucrose and ABA in maize endosperm. Plant Sci.

[60] Hoopes GM, Hamilton JP, Wood JC (2019). An updated gene atlas for maize reveals organ-specific and stress-induced genes. Plant J.

