# Supplementary material for: Insights into ZmWAKL in maize kernel development: genome-wide investigation and GA-mediated transcription
Source: BMC Genomics. 2023 Dec 11;24:760. doi: 10.1186/s12864-023-09849-6 (PMC10712088; doi:10.1186/s12864-023-09849-6)
Supplement: Supplementary file 1 — Additional file 1: Fig. S1. [file 12864_2023_9849_MOESM1_ESM.pdf]

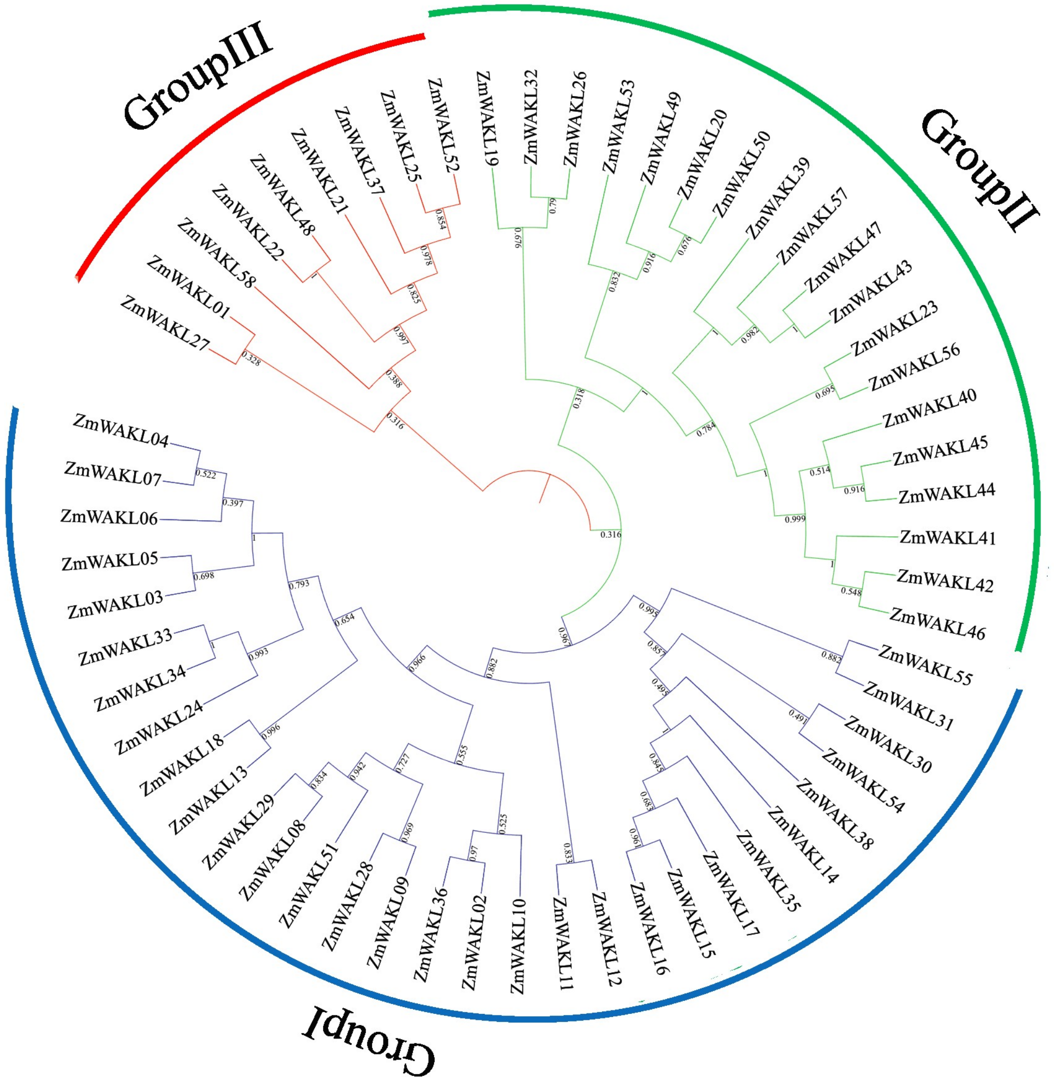

Fig.S1 Phylogenetic tree of ZmWAKL proteins with different color arcs representing distinct clusters of ZmWAKL group. Neighbor-joining (NJ) method with 1,000 bootstrap replicates was utilized to construct the unrooted phylogenetic tree based on the alignment of ZmWAKL domain sequences. The percentages of bootstrap numbers for the nodes are displayed on the branches.
