# Supplementary material for: Insights into ZmWAKL in maize kernel development: genome-wide investigation and GA-mediated transcription
Source: BMC Genomics. 2023 Dec 11;24:760. doi: 10.1186/s12864-023-09849-6 (PMC10712088; doi:10.1186/s12864-023-09849-6)
Supplement: Supplementary file 2 — Additional file 2: Fig. S2. [file 12864_2023_9849_MOESM2_ESM.pdf]

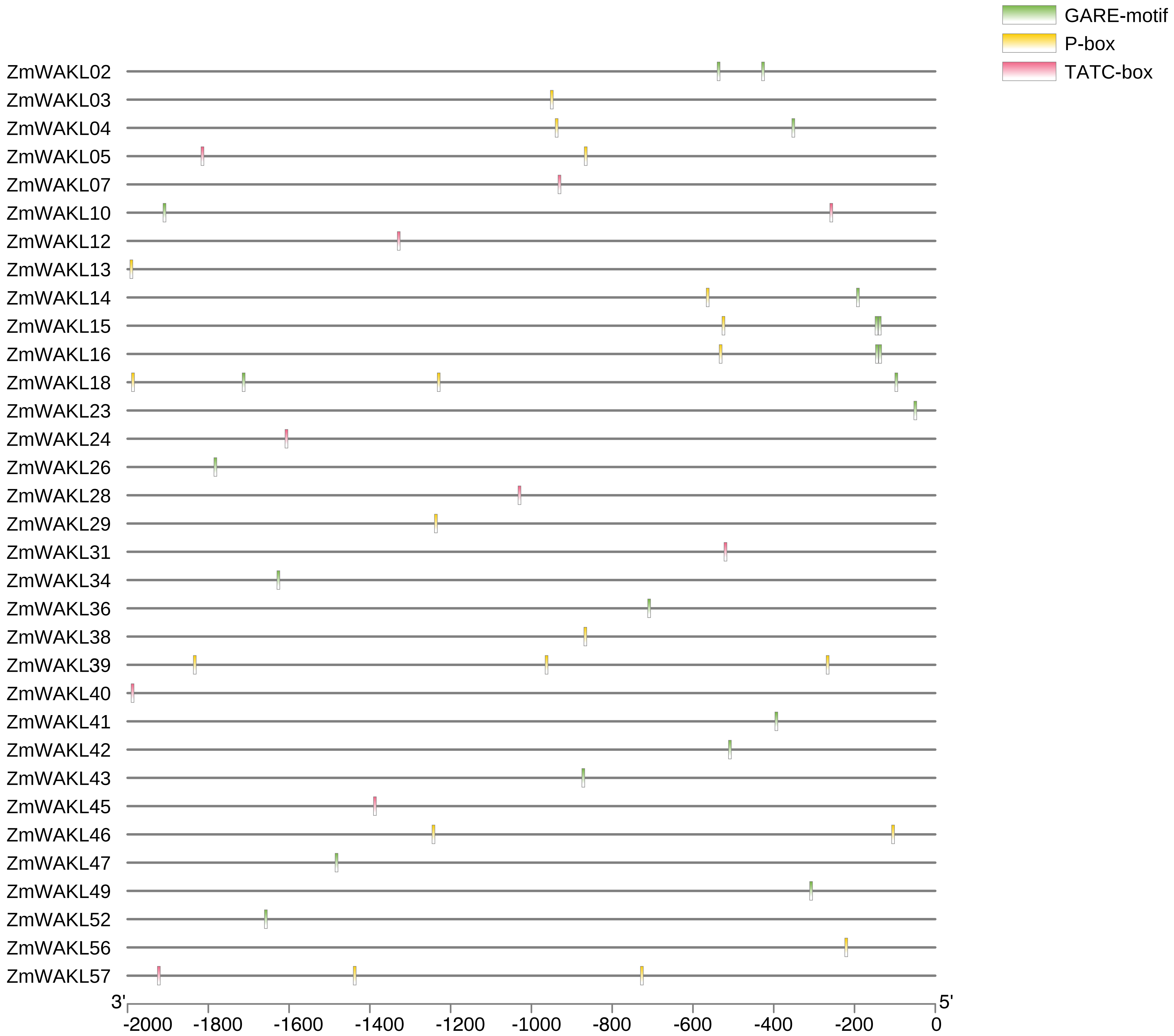

Fig.S2 Prediction of GA responsive related *cis*-acting elements in 33 *ZmWAKL* promoters. Promoter sequences (−2000 bp) of 33 *ZmWAKL* genes are analyzed by PlantCARE. The upstream length to the translation starts site can be inferred according to the scale at the bottom.
