# Supplementary material for: Insights into ZmWAKL in maize kernel development: genome-wide investigation and GA-mediated transcription
Source: BMC Genomics. 2023 Dec 11;24:760. doi: 10.1186/s12864-023-09849-6 (PMC10712088; doi:10.1186/s12864-023-09849-6)
Supplement: Supplementary file 3 — Additional file 3: Fig. S3. [file 12864_2023_9849_MOESM3_ESM.pdf]

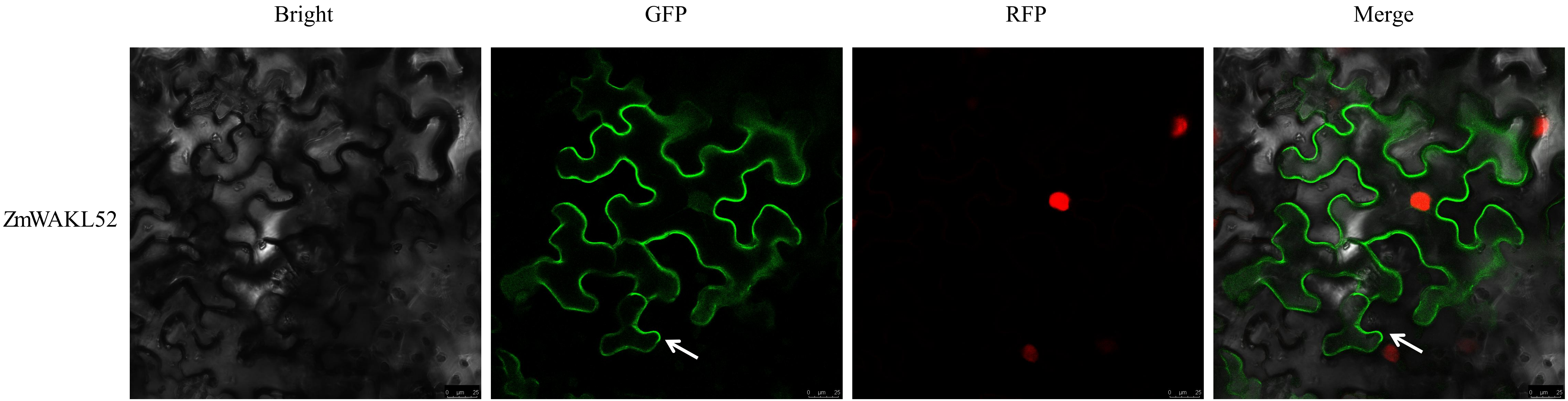

Fig.S3 Subcellular localization analysis of ZmWAKL52 conducted in *N. benthamiana* leaves. NLS-RFP were used as nuclear marker and show red fluorescence signals. The arrow indicates GFP signals present in cell wall. Scale bar = 25 μm.
